# Real-World Use of Anifrolumab for Articular Involvement in Systemic Lupus Erythematosus: A Monocentric Case Series and Systematic Review

**DOI:** 10.3390/jpm15110546

**Published:** 2025-11-10

**Authors:** Giulia Cassone, Filippo Santoro, Mariagrazia Nuara, Chiara Cabassi, Caterina Vacchi, Ottavio Secchi, Dilia Giuggioli

**Affiliations:** 1 Rheumatology Unit, Azienda Ospedaliero-Universitaria Policlinico di Modena, Via del Pozzo 71, 41125 Modena, Italyottavio.secchi@unimore.it (O.S.);; 2 Department of Medical and Surgical Sciences for Children and Adults, School of Medicine, University of Modena and Reggio Emilia, Via del Pozzo 71, 41125 Modena, Italy

**Keywords:** anifrolumab, systemic lupus erythematosus, articular involvement, real-world evidence, lupus arthritis, biologic therapy, personalized medicine

## Abstract

**Introduction:** This study evaluates the real-world application of anifrolumab in managing articular involvement in systemic lupus erythematosus (SLE), providing insights into its efficacy and safety in routine clinical practice. Additionally, a systematic review examines anifrolumab’s role specifically in joint manifestations of SLE, consolidating existing real-world data on its therapeutic impact in articular disease. **Methods:** This monocentric case series presents data from four patients with SLE-related arthritis treated with anifrolumab. Clinical outcomes, including joint symptoms, clinimetric indices (DAS28, SLEDAI-2K, and SLICC), and treatment tolerability, were assessed. Ultrasound evaluation did not represent an outcome since it was not performed regularly. A systematic review was conducted to explore anifrolumab’s real-world application in articular disease manifestations, offering a comparative perspective. **Results:** All patients achieved complete remission of arthritis and lupus disease activity within four months, with no serious adverse reactions and without treatment discontinuation. Additionally, two patients completely discontinued corticosteroid (GC) therapy within two months, while the remaining two significantly reduced their GC doses. Only three promising relevant articles emerged from the systematic review, underlining the need for further studies to better support the role of anifrolumab in the treatment of arthritis in SLE. **Conclusions:** These findings highlight anifrolumab’s practical utility in real-world settings, particularly for articular involvement, while the systematic review contextualizes its impact within SLE management. The results underscore anifrolumab’s potential as a valuable treatment option for joint manifestations of SLE, addressing an unmet clinical need in routine practice. This evidence may assist clinicians in selecting the most suitable therapeutic approach based on predominant clinical features, thus enhancing personalized treatment strategies in SLE.

## 1. Introduction

Systemic lupus erythematosus (SLE) is a chronic autoimmune condition characterized by heterogenous manifestations, ranging from mild to life-threatening. The broad spectrum of potential organ involvement varies from patient to patient, leading to diagnostic delays, significant challenges in disease management, and a frequent need for a multidisciplinary approach [1].

SLE arthritis treatment includes a limited number of treatment alternatives; in the case of an inadequate response to glucocorticoids (GCs) or hydroxychloroquine (HCQ), methotrexate is preferred for SLE arthritis, eventually associated with belimumab and or rituximab [2,3,4,5,6,7].

Recently, clinical trials demonstrated the efficacy and safety of a new biological agent, namely anifrolumab (TULIP-I and TULIP-II) [6,7], for treating extra-renal moderate-to-severe SLE. Anifrolumab is a fully human IgG1κ monoclonal antibody which inhibits all type I IFNs by binding to subunit 1 of the type I IFNAR (IFNAR1) with high affinity and specificity and reduces the levels of cell surface IFNAR1 available, inducing its internalization. Anifrolumab therefore inhibits IFN-responsive gene expression and downstream inflammatory and immunological processes and events.

Since 2021, it has been approved for SLE treatment in multiple countries.

Furthermore, the new EULAR guidelines underlined the need for sparing GC use and the possibility of early treatment with biological drugs even in patients with moderate disease [4,8].

In recent years, a number of clinical cases and case series describing the efficacy of anifrolumab in skin involvement have been published.

However, real-life data are limited, and its optimal use remains unclear, especially in domains other than cutaneous involvement.

We present a consecutive case series of SLE patients with long-standing disease and prevalent articular involvement treated with anifrolumab. This case series aims to underline how anifrolumab could represent a valid therapeutic opportunity even in case of severe joint involvement in SLE patients, with a reassuring safety profile.

Clinical outcomes, including joint symptoms, clinimetric indices (DAS28, SLEDAI-2K, and SLICC), and treatment tolerability, were assessed. Ultrasound evaluation did not represent an outcome since it was not performed regularly. Furthermore, a systematic review was conducted to explore anifrolumab’s real-world application in articular disease manifestations, offering a comparative perspective.

## 2. Case Series

### 2.1. Patient 1

A 49-year-old Cuban female was diagnosed with SLE in September 2023, presenting with alopecia, symmetrical polyarthritis, and mild pericardial effusion. Laboratory tests revealed neutropenia, lymphopenia, hypocomplementemia, and high-titer anti-dsDNA antibodies, consistent with active disease. The main clinical and serological features are summarized in Table 1. Joint ultrasound confirmed arthritis of the hands, wrists, and knees. The Systemic Lupus Erythematosus Disease Activity Index (SLEDAI) was 13, and the disease activity score 28 (DAS28) was 7.22.

Initial treatments with corticosteroids, HCQ, mycophenolate (MMF), and azathioprine (AZA) were discontinued due to intolerance or non-initiation due to personal choice.

During follow-up, laboratory analysis showed persistent disease activity (lymphopenia, complement consumption, high titer of anti-dsDNA). Clinical examination revealed active arthritis (TJ 5, SJ 2) and persistent alopecia.

In December 2023, monotherapy with anifrolumab 300 mg every four weeks was initiated, alongside monthly intravenous methylprednisolone (40 mg), aiming to improve compliance. The patient showed rapid clinical improvement, including a reduction in alopecia, arthritis flares, and pericardial effusion. Laboratory parameters normalized, and the SLEDAI decreased from 13 to 2. Glucocorticoids were discontinued.

After four months, arthritis of the wrists re-emerged. Methotrexate (MTX) was added, resulting in complete remission (DAS28: 2.18). A single dose of IV methylprednisolone was administered, and no further oral glucocorticoids were required.

### 2.2. Patient 2

A 38-year-old Caucasian female was diagnosed with SLE in 2018, with initial cutaneous involvement (subacute cutaneous lupus) and positive antinuclear antibody (ANA), anti-SSA/Ro, and anti-SSB/La antibodies. Anti-dsDNA was slightly positive in ELISA (14 UI/mL) yet negative in immunofluorescence assay. Hematological and immunological features, including complement levels and rheumatoid factor (RF) and anti-cyclic citrullinated peptide antibody (anti-CCP) positivity, are summarized in Table 1.

After years of stable disease, she developed inflammatory arthritis in May 2023, affecting the small joints of the hands, wrists, and right knee, with prolonged morning stiffness and elevated inflammatory markers. HCQ was discontinued due to intolerance (urticarial rash), and MTX 15 mg/week was initiated in June 2023 following a diagnosis of Rhupus syndrome, with partial improvement.

In February 2024, she experienced a flare of arthritis, confirmed by joint ultrasound. The SLEDAI was 6, and the DAS28 was 5.59. Anifrolumab 300 mg IV every four weeks was added to MTX in March 2024, along with a short course of glucocorticoids.

Following the second infusion, the patient achieved complete clinical remission: normalization of complement levels, negativization of anti-dsDNA, and resolution of joint symptoms (DAS28: 1.89; SLEDAI: 1). She reported no residual arthralgia or morning stiffness and was able to discontinue steroid therapy entirely.

### 2.3. Patient 3

A 41-year-old Caucasian female with long-standing SLE, diagnosed in 2008, presented with relapsing arthritis and cutaneous manifestations. Her medical history and key clinical and serological features are summarized in Table 1.

Immunological tests showed ANA and anti-SSA positivity, with normal complement levels and negative anti-dsDNA, RF, and anti-CCP.

Over the years, she received multiple conventional and biologic drugs (leflunomide, sulfasalazine, ciclosporin, MTX, rituximab, tocilizumab, belimumab, baricitinib, and abatacept), with limited efficacy or intolerance. Chronic GC therapy (4–16 mg/day methylprednisolone) led to significant damage, including osteonecrosis (medial femoral condyle and left ankle) and osteoporosis with multiple vertebral fractures (D13, L3, L4). Her SLICC/ACR damage index score was 6.

In October 2023, following a flare of joint and skin disease (SLEDAI: 6; DAS28: 4.57), MTX was increased to 15 mg/day, and anifrolumab 300 mg IV monthly was initiated. Over the subsequent 14 months, the patient remained flare-free, with sustained remission (SLEDAI: 0; DAS28: 2.31). The new regimen allowed stabilization of glucocorticoid therapy at 4 mg/day without further escalation.

### 2.4. Patient 4

A 42-year-old African female with SLE diagnosed in 2009 was admitted to our department in 2015. Her clinical history included hematological abnormalities (lymphopenia, neutropenia, macrocytic anemia), arthritis, malar rash, and lupus nephritis (class III), with immunological positivity for ANA, anti-Sm, anti-dsDNA, and anti-RNP70, along with low complement levels. Key clinical and serological features are summarized in Table 1.

She had previously received multiple immunosuppressive therapies, including HCQ, MMF, cyclosporin, cyclophosphamide, rituximab, and belimumab, with limited efficacy and poor tolerability. Her medical history was further complicated by occult HBV infection, Strongyloides stercoralis, and Enterococcus faecium meningitis. Poor compliance and frequent loss to follow-up were documented.

In September 2023, after a prolonged interruption of care, she was hospitalized for reassessment. Despite clinical remission (SLEDAI: 0), signs of cortisolism were evident due to self-administered methylprednisolone (≥8 mg/day). Given her steroid dependence and prior treatment failures, anifrolumab 300 mg IV monthly was initiated in November 2023.

After four months, clinical remission was maintained, and glucocorticoid therapy was successfully tapered to 2 mg/day. Unfortunately, since March 2024, the patient has not maintained regular follow-up.

## 3. Summary of Clinical Data

Our four patients were selected consecutively from patients referred to our Rheumatology Unit between September 2023 and March 2024, based on the presence of active articular involvement and indication for biologic therapy.

All patients fulfilled the available classification criteria for SLE.

On average, all patients in our series were female, with a mean age of 48.25 (range 56–38) and a median disease duration of 108.75 months (range 192–15). All patients had articular involvement with proven polyarticular arthritis.

One patient displayed erosive rheumatoid-like arthritis documented by X-ray, while another reported overlap syndrome (Rhupus syndrome) with positivity to RF and anti-CCP antibodies.

One patient had been recently diagnosed with SLE, while another had a disease duration of less than 5 years. The remaining two patients had long-standing SLE with a disease duration exceeding 10 years.

Two patients were intolerant to HCQ. Three patients received anifrolumab associated with MTX. For two patients, GC therapy was discontinued after less than 2 months following anifrolumab therapy. These patients had a short disease duration. The other two patients with long-standing disease were able to taper GC therapy to low doses (4 mg and 2 mg of methylprednisolone per day) in just a few months.

Two patients previously received rituximab and belimumab; the latter drug was suspended for ineffectiveness in both cases. One patient was also previously treated with other off-label biologic and targeted synthetic disease modifying anti rheumatic drugs (bDMARDs and tsDMARDs) to try to control arthritis flare-ups.

No serious adverse reactions were observed, and no patients discontinued anifrolumab.

The mean follow-up was 9.75 months, and patient 3 underwent the longest treatment (14 months).

Before the introduction of anifrolumab therapy, the mean SLEDAI of our patients was 6.25 (moderate disease relapse), and the DAS28 was 5 (high disease activity).

All patients experienced remission of arthritis as well as SLE after less than 4 months (mean SLEDAI 0.75; mean DAS28 2.19). The clinical baseline characteristics of our patient series are summarized in Table 1, while outcome data—including disease activity scores, treatment response, and glucocorticoid tapering—are presented in Table 2.

## 4. Review of the Literature

### 4.1. Methods

The study protocol was developed according to the Preferred Reporting Items for Systematic Reviews and Meta-Analyses (PRISMA) guidelines (the PRISMA checklist is available in the Supplementary Table S2). Studies were selected according to the Patients–Exposure–Outcome (PEO) framework for eligibility regarding the research question outlined in the Supplementary Material (Supplementary Table S1).

PubMed and Embase were employed to retrieve relevant publications up until the 8 June 2025. The search query used was the following: anifrolumab AND (joint OR arthritis). In order to be included in the review, the publications had to have the following characteristics: (1) real-life studies, including case series or case reports; (2) describe articular involvement before initiating treatment with anifrolumab, meaning patients with clinical or ultrasound-proven arthritis; and (3) describe the articular outcomes after initiation with anifrolumab, either clinical (e.g., resolution of clinical arthritis) or through ultrasound examination. Congress abstracts, without a corresponding full-text publication, were included provided that detailed data were reported. Due to their limited methodological detail, these sources were interpreted with caution.

We only considered articles in English. Two screening rounds were performed. In the first round, two reviewers (F.S. and G.C.) evaluated, in duplicate, titles and abstracts in terms of relevance to the review. In the second round, full texts of the articles included during the first round were retrieved and re-assessed for eligibility. Data regarding articular involvement, concomitant therapies, previous therapies, use of steroids, and clinical outcomes were collected.

Due to the small number of eligible studies and the limited strength of the underlying clinical evidence, this systematic review was not prospectively registered in PROSPERO. Moreover, no formal quality assessment of the included studies was performed, which represents a methodological limitation of the systematic review.

### 4.2. Results

**Figure 1:**
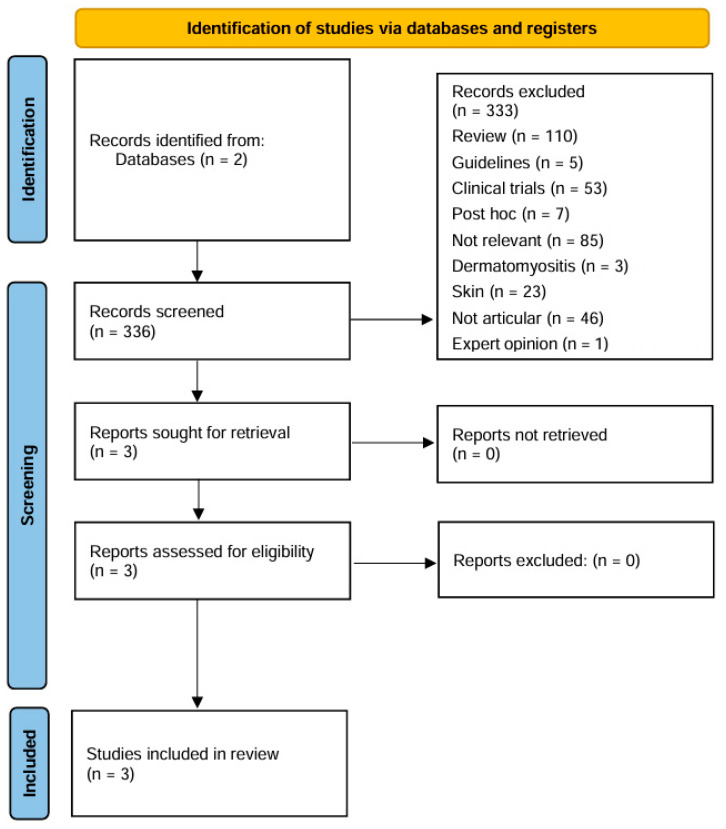
The main characteristics of the studies are summarized in Table 3.

In total, 66 results were found on PubMed, and 270 results were found on Embase, for a total of 336 articles. A detailed flow chart describing the study inclusion and exclusion process is shown in Figure 1. Overall, out of 336 articles, only 3 papers fulfilled the criteria to be included in this review.

Overall, a total of 31 patients with articular involvement are described in the available literature. The diagnosis was clinical in most patients, with only three cases that were ultrasound-proven [9]. All the patients had already failed at least one conventional DMARD. Many patients had already undergone failed treatment with belimumab, with at least 3 cases described by Ceccarelli et al. [9] and 20 patients described by Tani et al. [10]. Some patients had also undergone other failed biological therapies, mainly rituximab.

Complete resolution of arthritis was seen in 17 cases out of 31 cases described (55% of patients). Regarding the steroid-sparing effect of anifrolumab, in the available case series, a reduction in steroid treatment is only described in the series by Maicas et al., with all patients (n = 3 patients) that were able to reduce the prednisone dose. In their prospective study, Tani et al. described a statistically significant effect in reducing steroid therapy at one month (7.6 ± 5.0 mg/day vs. 6.4 ± 4.9 mg/day, *p* = 0.04).

**Table 3:** Summary of available data for the systematic review of the literature.

Author, Year	Type of Article	Number of SLE Patients	Ultrasound-Proven	Outcome	Concomitant Treatment
Maicas, 2024 [11]	Case series	3 patients	No	Resolution of arthritis in 2 patients	Not specified
Tani, 2024 [10]	Multicenter, prospective	25 patients	No	Resolution of arthritis in 13 patients; significant reduction in TJ/SJ count at 4 months (*p*: 0.026), 12 months (*p*: 0.021), and 24 months (*p*: 0.012)	NA
Ceccarelli, 2025 [9]	Case series	3 patients	Yes	Reduction in DAS 28, US synovitis score, and US PD score in all patients	HCQ (3 pts), MTX (2 pts), PDN 10 mg/daily (3 pts)

Legend: DAS 28 = disease activity score in 28 joints; HCQ = hydroxicloroquine; MTX = methotrexate; PD = power Doppler; PDN = prednisone; TJ/SJ = tender joint/swollen joint; US = ultrasound; NA = not available.

## 5. Discussion

Systemic lupus erythematosus is a heterogeneous autoimmune disorder, with diverse manifestations, among which articular involvement is both frequent and impactful in terms of patient quality of life. While randomized trials such as MUSE and TULIP-2 [12,13] have demonstrated the efficacy of anifrolumab, particularly in cutaneous disease and steroid tapering, its real-world use remains insufficiently explored in routine clinical practice [10,14].

Real-world evidence has primarily focused on cutaneous lupus [15,16], with relatively limited data on systemic manifestations. Notable exceptions include reports of benefits in lupus nephritis [17,18] and neuropsychiatric lupus [19] and steroid-sparing effects in refractory disease, as seen in the LOOPs registry [14] and select cohorts [10,19]. However, evidence supporting its use in articular disease specifically is scarce.

Articular involvement, ranging from inflammatory arthralgias to overt erosive arthritis, is one of the most frequent manifestations of SLE [20], with a major impact on quality of life and disease burden [21]. Despite its prevalence, treatment is often empirical, with limited data supporting cDMARDs. Belimumab has shown efficacy in reducing joint disease activity [22], yet its ability to prevent progressive joint damage remains unclear. Given the limitations of current therapies, anifrolumab offers a targeted approach, potentially improving SLE-related arthritis management. Post hoc analyses of TULIP-1 and TULIP-2 demonstrated significant improvements in the articular domain, but real-world data remain scarce [9,10,11].

Our monocentric case series contributes to closing this gap, showing complete arthritis remission (both clinical and by DAS28) in all four patients within four months, with a mean SLEDAI reduction from 6.25 to 0.75 and DAS28 from 5.0 to 2.19. These results appear more favorable than those reported in TULIP trials, where the arthritis response rates were 8.2% (SLEDAI), 11.8% (BILAG), and 12.6% (joint response). The higher remission rate observed in our study may be attributed to differences in patient profiles, including prior exposure to conventional and biologic DMARDs, the concomitant use of MTX in three patients, or a shorter disease duration, which could have influenced treatment responsiveness. It should also be noted that the arthritis endpoints used in the TULIP trials differ from the DAS28 and SLEDAI definitions adopted in our study, which may further limit direct comparability between trial and real-world outcomes.

Among the limitations of our study is the lack of systematic ultrasound assessment, which—while not routinely performed in our cohort—has been employed in other studies [9] to validate clinical joint response and detect subclinical synovitis. Furthermore, our case series includes exclusively female patients. Musculoskeletal involvement in SLE has indeed been reported to occur less frequently in males, but this sex imbalance can represent a limitation in the evaluation of the efficacy of anifrolumab.

Finally, the systematic review conducted as part of this study identified only three relevant articles, highlighting the currently limited evidence base regarding anifrolumab’s use in articular manifestations of SLE.

Our findings align with those of Tani et al.’s [10] multicenter study and the recent case series by Ceccarelli et al. [9], both supporting a rapid and sustained articular response in routine care. Tani et al. reported significant improvements in tender (*p* = 0.026) and swollen (*p* = 0.017) joint counts, while our systematic review showed that 55% of patients with articular involvement achieved clinical remission—results that are consistent with those observed in our cohort.

The role of MTX co-treatment deserves attention. While its immunomodulatory effect may enhance therapeutic response, data remain sparse. In our cohort, three out of four patients received MTX in combination, which may have contributed to the rapid remission observed. Namely, in the first case, MTX was added to sustain the clinical response to anifrolumab, while in the second and third cases, MTX alone was ineffective or poorly tolerated. Interestingly, in the Ceccarelli series, two out of three patients also received MTX, suggesting potential synergy worth further exploration.

Taken collectively, these cases highlight the potential limitations of MTX both in terms of variable effectiveness and tolerability. Although MTX co-treatment may have amplified the effect in some cases, similar results in patients without MTX suggest independent efficacy. Future research is necessary to determine if MTX is crucial for achieving therapeutic success in this clinical context, potentially through a synergistic effect with anifrolumab through mechanisms such as modulation of T-cell activation or cytokine suppression.

However, the potential benefits must be balanced against the added risk of adverse events, particularly in the presence of complex comorbidities.

In our cohort, both early (<5 years) and long-standing (>10 years) SLE patients achieved arthritis remission within four months, suggesting that the disease duration did not influence the response. This mirrors findings from post hoc TULIP analyses and supports the efficacy of anifrolumab across disease stages.

Importantly, glucocorticoid tapering was achieved in all patients: two discontinued entirely, and two reduced doses to below 5 mg/day. These results underline anifrolumab’s potential steroid-sparing effect, as also highlighted by Maicas et al. [11] and Tani et al. [10].

Given the well-known risks of long-term glucocorticoid exposure (osteonecrosis, osteoporosis, and organ damage), this aspect holds substantial clinical value. Had biologic therapy been introduced earlier in our two long-standing SLE patients, it is conceivable that complications such as vertebral fractures or erosive damage might have been mitigated.

No serious adverse events were reported in our cohort, and no patients discontinued therapy. This is consistent with the TULIP trials, where the most common adverse events included upper respiratory infections, bronchitis, and herpes zoster. Our findings confirm anifrolumab’s favorable safety profile, reinforcing its real-world tolerability, particularly in patients with lupus arthritis, where long-term safety data remain limited.

Although anifrolumab has demonstrated promising results in SLE-related arthritis, several aspects warrant further investigation to optimize its therapeutic use. In particular, its long-term impact on joint damage progression, especially in patients with Jaccoud’s arthropathy or subclinical erosions, remains unknown. Broader real-world studies could clarify its potential disease-modifying properties and inform structural outcome measures. The possible synergistic role of methotrexate, suggested by our cohort and previous reports, should also be explored, along with whether early versus long-standing disease modulates the treatment response.

Despite articular involvement being one of the most prevalent and disabling features of SLE, real-world data on anifrolumab in this context are still limited. Our findings help address this gap and offer valuable real-world insights. However, given the limited sample size and the scarcity of robust literature on this topic, they should be interpreted as exploratory. High-level evidence studies, namely randomized or double-blind placebo-controlled trials or larger prospective cohort studies, are warranted to validate these findings, identify predictors of response, and clarify how anifrolumab can be optimally integrated into individualized treatment approaches. By tailoring therapeutic decisions to each patient’s dominant clinical phenotype, our findings may support more personalized and effective management of lupus arthritis. As evidence continues to grow, anifrolumab may become a pivotal option for targeted disease control and steroid minimization in a population with a substantial unmet clinical need.

## Figures and Tables

**Table 1:** Baseline characteristics of SLE patients treated with anifrolumab.

	Gender, Ethnicity	Age	Diagnosis	Autoimmunity	Comorbidities	Organ Involvement at Diagnosis	Previous Therapies	Treatment Intolerance	Outcome
Pt. 1	F, Cuban	49	2023	anti-U1RNP, anti-SSA/Ro, anti-dsDNA	None	Alopecia, inflammatory arthralgias, symmetrical polyarthritis, sicca syndrome, neutropenia, and lymphopenia	GCs, HCQ, MMF, AZA	HCQ, MMF, AZA	Persistent alopecia, lymphopenia, complement consumption, high titer of anti-dsDNA, active arthritis
Pt. 2	F, Caucasian	38	2018	anti-SSA/Ro, anti-SSB/La, anti-dsDNA (ELISA), FR, anti-CCP	None	Subacute LE and arthritis	GCs, HCQ, MTX	HCQ	Arthritis relapse
Pt. 3	F, Caucasian	56	2007	anti-SSA	Hepatic steatosis, multinodular thyroid goiter, prolactin-secreting pituitary microadenoma	Raynaud phenomenon, arthritis, and subacute LE	GCs, HCQ, LEF, SLZ, CIC, MTX; RTX, TOCI, BARI, BELI	CIC, MTX, RTX, TOCI	Recurrent joint and skin manifestations; GC dependence with side effects (SLICC 6)
Pt. 4	F, African	50	2009	anti-Sm, anti-RNP70, anti-dsDNA	Occult HBV infection, Strongyloides stercoraris infection, Enterococcus faecium meningitis	Articular, cutaneous, hematological, and renal	GCs, HCQ, MMF, CIC, CFX; RTX, BELI	None; poor compliance	GC dependence with signs of cortisolism

Legend: LE = lupus erythematosus; HCQ = hydroxychloroquine; MMF = mycophenolate mofetil; AZA = azathioprine; LEF = leflunomide; SLZ = sulfasalazine; CIC = cyclosporin; MTX = methotrexate; RTX = rituximab; TOCI = tocilizumab; BARI = baricitinib; BELI = belimumab; GCs = glucocorticoids.

**Table 2:** Outcomes of SLE patients treated with anifrolumab.

	Disease Duration (Months) *	Arthritis (Characteristics)	Erosive Arthritis	Other Organ Involvement *	Concomitant Therapy *	Duration of ANI Therapy (Months)	SLEDAI before ANI	SLEDAI Last Clinical Evaluation	DAS28 before ANI	DAS28 Last Clinical Evaluation	GCs Last Clinical Evaluation	Side Effects	Response
Pt. 1	15	Symmetrical polyarthritis	No	Cutaneous, Hematological, and serositis	GCs (40 mg IV monthly during the first two infusions)	12	13	2	7.22	2.18	0	None	Yes
Pt. 2	60	Symmetrical polyarthritis	No (Rhupus)		MTX, GCs (8 mg)	9	6	1	5.59	1.89	0	None	Yes
Pt. 3	192	Symmetrical polyarthritis	Yes	Cutaneous	HCQ, MTX, GCs (6 mg)	14	6	0	4.57	2.31	4 mg	None	Yes
Pt. 4	168	Polyarthritis	No	Cutaneous	GCs (8 mg)	4	0	0	2.38	2.38	2 mg	None	Yes

* when anifrolumab was started. Legend: ANI = anifrolumab; HCQ = hydroxychloroquine; MTX = methotrexate; GCs = glucocorticoids; DAS28 = disease activity score in 28 joints; SLEDAI = Systemic Lupus Erythematosus Disease Activity Index.

## Data Availability

The data presented in this study are available on request from the corresponding author.

## Supplementary Materials

The following supporting information can be downloaded at https://www.mdpi.com/article/10.3390/jpm15110546/s1, Table S1: Quality assessment of diagnostic accuracy studies (QUADAS) for articles included in the systematic review. Table S2: PRISMA Checklist.
